# Extended Target Tracking and Feature Estimation for Optical Sensors Based on the Gaussian Process

**DOI:** 10.3390/s19071704

**Published:** 2019-04-10

**Authors:** Haoyang Yu, Wei An, Ran Zhu

**Affiliations:** 1 College of Electronic Science and Technology, National University of Defense Technology, Changsha 410073, China; anwei@nudt.edu.cn; 2 Institute of Systems Engineering, AMS, PLA, Beijing 100141, China; zhuran@nudt.edu.cn

**Keywords:** extended target tracking, amplitude information, Gaussian process, target feature estimation

## Abstract

A problem of tracking surface shape-shifting extended target by using gray scale pixels on optical image is considered. The measurement with amplitude information (AI) is available to the proposed method. The target is regarded as a convex hemispheric object, and the amplitude distribution of the extended target is represented by a solid radial function. The Gaussian process (GP) is applied and the covariance function of GP is modified to fit the convex hemispheric shape. The points to be estimated on the target surface are selected reasonably in the hemispheric space at the azimuth and pitch directions. Analytical representation of the estimated target extent is provided and the recursive process is implemented by the extended Kalman filter (EKF). In order to demonstrate the algorithm’s ability of tracking complex shaped targets, a trailing target characterized by two feature parameters is simulated and the two feature parameters are extracted with the estimated points. The simulations verify the validity of the proposed method with compared to classical algorithms.

## 1. Introduction

The extended target tracking (ETT) problem has been widely studied to estimate target motions and shape. With the development of the sensors, more information such as amplitude, color, etc. can be collected. This additional information should be fully utilized since it helps to improve the tracking precision and contains more abundant target characteristics. Therefore, new demand for an effective algorithm that can take advantage of additional information has emerged.

Many scholars have carried out research on ETT problems from different aspects such as shape modeling, measurement modeling and dynamic modeling. In this letter, we focus on the first aspect, i.e., the shape model method for an extended object. The current research achievements can be divided into the following three categories, corresponding to three levels of shape complexity. **No extent**: the simplest approach is to ignore the target extent. Under the assumptions that the measurements are distributed in the neighbor of target with a Poisson distributed number, Gaussian mixture probability hypothesis density (GM-PHD) filter is proposed [1,2]. **Simple extent**: a better modeling approach is to assume the target as a basic geometry, such as an ellipse, a circle or a rectangle. The most widely adopted method is based on a random matrix (RM), which represents the target extent as an ellipse [3,4,5,6,7]. For the target with non-elliptic shapes, a combination of multiple elliptical subobjects can be employed to approximate its real appearances [8,9,10]. Rectangle shape is usually adopted when tracking cars and other box-shaped objects [11,12,13]. **Arbitrary extent**: a more accurate and general approach is called random hypersurface models (RHMs) [14,15,16], which models the extent as a star-convex shape [17]. The RHMs can adapt to more flexible shapes in contrast with RM. The Gaussian process (GP) method can also be employed to estimate a target with highly nonlinear extent [18,19]. A more complete review for an ETT problem about shape model methods is presented in [20]. As mentioned in part **Simple extent**, the classical RM, RHMs and other variations only output the two-dimensional contour of the target, they do not have the ability to utilize extra information while the GP has the potential to realize that. The GP based method can theoretically estimate target of any dimension due to the fitting ability for highly nonlinear function.

GP has been adopted to learn the shape of a stationary three-dimensional object in [21,22], but none of them provide a recursive solution in a tracking application. A recursive solution for a cube-shaped object is provided in [23] by using point measurements collected from the target surface, but the target shape is relatively simple and there are no comparisons to the classical methods. In this letter, we extend the GP method to the problem of ETT on an image plane for optical sensors. Since the traditional RM, RHMs and GP are unable to utilize the amplitude information, we try to present a novel approach to overcome this problem. The surface of an extended target is described with solid radial function, rather than a two-dimensional counter. In order to adapt to the three-dimensional target tracking, the spherical difference was adopted rather than the angle difference of two two-dimensional angles. The RM and RHMs need to parameterize the target extent and estimate these parameters, while this letter estimates some selected points on the target surface. These selected points can represent the target distribution feature. Thus, the proposed method does not require any parametric model. The measurements with amplitude are converted into polar coordinates, and the measurements are used to estimate these points by the GP method with modified covariance function. In addition, we use the estimated points to extract the feature parameters of the target distribution, which are beneficial to the identification and classification of the target in the future work.

## 2. GP Theory

The GP is a kind of stochastic process in probability theory and mathematical statistics, which combines a series of random variables that follow normal distribution within an index set. Unknown complicated functions can be learned with training data. In this section, GP theory is directly applied to tracking application. More detail about the theory can be referred to [18].

### 2.1. Basic GP

GP is a typical stochastic process. it is always used to model spatially correlated measurements [24]. With *t* being input, the distribution of a GP f(t) can be uniquely determined by its mean function μ(t) and covariance function $k(t,t^{\prime })$ as

(1) $$\mu \left(t\right)=E\left[f(t)\right],$$

(2) $$k\left(t,t^{\prime }\right)=E\left[\left(f(t)-\mu (t)\right){\left(f\left(t^{\prime }\right)-\mu \left(t^{\prime }\right)\right)}^{T}\right].$$

Then, the GP can be marked as

(3) $$f\left(t\right)∼GP\left(\mu \left(t\right),k\left(t,t^{\prime }\right)\right).$$

For the function value of the finite input $t_{1}\cdots t_{N}$, the Gaussian process is a generalization of the multivariate Gaussian probability distribution, i.e.,

(4) $$\left[\begin{array}{c} f(t_{1})\\ \vdots \\ f(t_{N}) \end{array}\right]∼N\left(\mu ,K\right),$$

where

(5) $$\mu =\left[\begin{array}{c} \mu (t_{1})\\ \vdots \\ \mu (t_{N}) \end{array}\right],\;K=\left[\begin{array}{ccc} k(t_{1},t_{1}) & \cdots & k(t_{1},t_{N})\\ \vdots & & \vdots \\ k(t_{N},t_{1}) & \cdots & k(t_{N},t_{N}) \end{array}\right].$$

### 2.2. GP Regression

**Figure 1:**
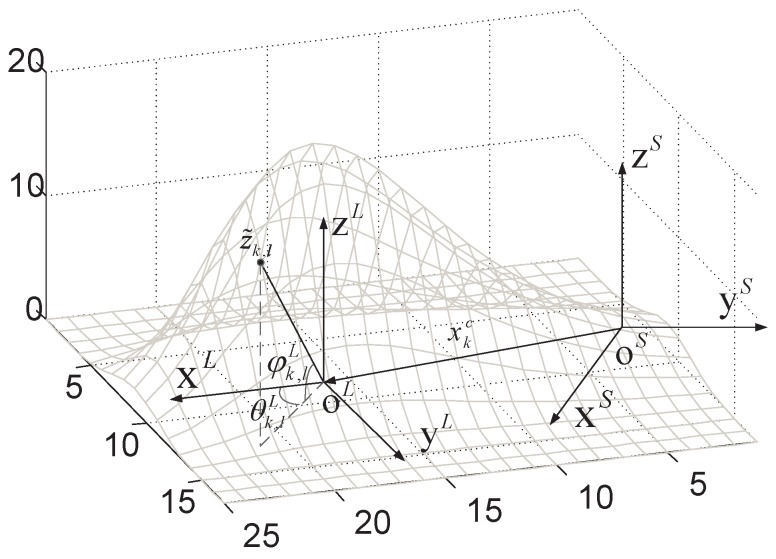
Sketch of the target surface described by a solid radial function.

The distribution of a highly nonlinear function can be fitted with a Gaussian process regression (GPR) method. In this subsection, the GPR is adopted to model the target extent. In tracking application, the function value $f={\left[f\left(t_{1}^{f}\right),\cdots ,f\left(t_{N}^{f}\right)\right]}^{T}$ for the corresponding input $t^{f}={\left[t_{1}^{f},\cdots ,t_{N}^{f}\right]}^{T}$ is learned by using the measurement $z={\left[z_{1},\cdots z_{M}\right]}^{T}$ and the corresponding input $t={\left[t_{1},\cdots t_{N}\right]}^{T}$. For a target with amplitude information shown in Figure 1, the above measurement z is defined by $\left\{r_{z},c_{z},I_{z}\right\}$, where $r_{z}$, $c_{z}$ and $I_{z}$ represent the row, column, and amplitude of the measurement in grayscale image, respectively. In our application, the input t=ψ, where $\psi \overset{\Delta }{=}\left(\theta ,\varphi \right)$ is the azimuth and pitch angle of a solid angle. The radius value $z_{k}$ and the function value $f=f(\psi_{k})$ satisfy the joint Gaussian distribution

(6) $$\left[\begin{array}{c} z_{k}\\ f \end{array}\right]∼N\left(0,\left[\begin{array}{cc} k(\psi_{k},\psi_{k})+R & K(\psi_{k},\psi^{f})\\ K(\psi^{f},\psi_{k}) & K(\psi^{f},\psi^{f}) \end{array}\right]\right).$$

According to the GP theory, the likelihood and initial priori probabilities are given as follows:

(7) $$p(\left.z_{k}\right|f)=N\left(z_{k};H_{k}^{f}f,R_{k}^{f}\right),$$

(8) $$p(f)=N\left(0,P_{0}^{f}\right),$$

where

(9) $$H^{f}\left(\psi_{k}\right)=K\left(\psi_{k},\psi^{f}\right){\left[K(\psi^{f},\psi^{f})\right]}^{-1},$$

(10) $$R^{f}\left(\psi_{k}\right)=k\left(\psi_{k},\psi_{k}\right)+R-K\left(\psi_{k},\psi^{f}\right){\left[K(\psi^{f},\psi^{f})\right]}^{-1}K\left(\psi^{f},\psi_{k}\right),$$

(11) $$\begin{array}{cc} P_{0}^{f}=K\left(\psi^{f},\psi^{f}\right) & \psi^{f}=\left(\theta^{f},\varphi^{f}\right). \end{array}$$

### 2.3. Recursive GPR

In order to realize the real-time recursive processing, the approximate method is usually used to solve the recursive problem. Bayes formula is applied to the posterior probability $p\left(f\left|z_{1:N}\right.\right)$, which is

(12) $$\begin{array}{cc} p\left(f\left|z_{1:N}\right.\right) & \propto p\left(z_{N}\left|f,z_{1:N-1}\right.\right)\cdot p\left(f\left|z_{1:N-1}\right.\right),\\ & \propto \prod_{k=1}^{N}p\left(z_{k}\left|f,z_{1:k-1}\right.\right)\cdot p\left(f\right). \end{array}$$

Therefore, it can be approximated that f is independent from the previous measurement $z_{1:k-1}$, which means that f is completely statistically independent from the previous measurement, i.e.,

(13) $$p\left(z_{k}\left|f,z_{1:k-1}\right.\right)=p\left(z_{k}\left|f\right.\right).$$

### 2.4. Covariance Function Modification

In the covariance function used in Ref. [18], the squared Euler distance between the two plane angles is used to measure the correlation, i.e.,

(14) $$k\left(\psi ,\psi^{\prime }\right)=\sigma_{f}^{2}\exp\left(-\frac{d(\psi ,\psi^{\prime })}{2l^{2}}\right)+\sigma_{r}^{2},$$

(15) $$d\left(\psi ,\psi^{\prime }\right)={∥\psi -\psi^{\prime }∥}^{2},$$

where $\sigma_{f}^{2}$ denotes the variance of the measurement amplitude and *l* denotes the scale of the function; ${∥\cdot ∥}^{2}$ denotes the squared Euler distance. Since the target amplitude surface is considered as a convex hemisphere, it is obvious that the spherical distance is more reasonable to measure the correlation of the two solid angles, which is expressed as

(16) $$d\left(\psi ,\psi^{\prime }\right)=\arccos{\left[\cos\left(\varphi \right)\cos\left(\varphi^{\prime }\right)\cos\left(\theta -\theta^{\prime }\right)+\sin\left(\varphi \right)\sin\left(\varphi^{\prime }\right)\right]}^{2}.$$

## 3. EKF Model

Kalman filters such as EKF and Unscented Kalman filter (UKF) [25,26] are the most commonly used filters for nonlinear filtering. Since the EKF exerts lower computing complexity than the UKF, we employed EKF as the Bayesian approximation in this section.

### 3.1. Measurement Amplitude Modification

Since the measurement amplitude and target size are different in units as well as unequal in the values, in order to obtain a better extent estimation, we hope that the input $x^{f}$ can be distributed as uniformly as possible on the target surface. Therefore, the measurement amplitude and target size should be close in order of magnitude, which means that the amplitude information needs to be reasonably modified. Let $S_{k}$ represent the number of pixels covered by the target, and let $I_{z_{k}}$ denote the amplitude value of all the target measurements collected from the optical image plane. Then, we define a modification factor β as

(17) β=SkSkmaxIzkmaxIzk.

With the amplitude value of the measurements multiplied by the modification factor β, the maximum value of the modified measurement $\max\left(I_{z_{k}}^{\prime }\right)=\beta \cdot \max\left(I_{z_{k}}\right)=\sqrt{S_{k}}$. It can be seen that, after modification, the amplitude value $I_{z_{k}}^{\prime }$ and the target scale $\sqrt{S_{k}}$ are at the same order of magnitude. Thus, the modified measurement is re-defined as

(18) $${\tilde{z}}_{k}\overset{\Delta }{=}\left\{r_{z_{k}},c_{z_{k}},\beta I_{z_{k}}\right\},$$

where $r_{z_{k}}$, $c_{z_{k}}$ and $I_{z_{k}}$ represent the row, column, and amplitude of the measurement $z_{k}$. We now replace $z_{k}$ with the modified measurement ${\tilde{z}}_{k}$ for the rest of this letter.

### 3.2. Measurement Model with Amplitude

With the amplitude information considered, the target dimension is augmented to three, and the target extent is regarded as a hemispherical convex surface. Thus, the extent of the target can be represented by a function of the solid angle ψ=(θ,φ) and radius r=f(ψ) in polar coordinates. Two coordinate systems are adopted. The sensor coordinate system is fixed to the sensor and local coordinate system is fixed to the body of the extended target, denoted by *S* and *L*, respectively. The origin of the local system $o^{L}$ is combined with the mass center of the target and fixed on the image plane; the $x^{L}$ axis lies in the image plane and points to the target moving direction, the $z^{L}$ axis is perpendicular to the plane. $x^{L}$, $y^{L}$ and $z^{L}$ follow the right-hand rule. The target extent is represented in the local coordinate system and the target center is presented in the sensor coordinate system.

As is shown in Figure 1, the target measurement ${\tilde{z}}_{k,l}$ can be expressed in terms of the direction vector $p(x_{k}^{c})$ and radius $f(\psi_{k,l}^{L}\left(x_{k}^{c},\zeta_{k}\right))$ relative to the centroid $x_{k}^{c}$, which is

(19) $${\tilde{z}}_{k,l}=x_{k}^{c}+p\left(x_{k}^{c}\right)f\left(\psi_{k,l}^{L}\left(x_{k}^{c},\zeta_{k}\right)\right)+{\bar{e}}_{k,l},$$

where ${\bar{e}}_{k,l}∼N\left(0,R\right)$, and $p\left(x_{k}^{c}\right)=p\left(\psi_{k,l}^{S}\left(x_{k}^{c}\right)\right)=\frac{{\tilde{z}}_{k,l}-x_{k}^{c}}{\left|\right|{\tilde{z}}_{k,l}-x_{k}^{c}\left|\right|}$ is the unit vector that points to the measurement ${\tilde{z}}_{k,l}$ from centroid $x_{k}^{c}$.

Reviewing the likelihood given in Equation (7), $H_{k}^{f}f$ is the mean of the random variable $z_{k}$ and $R_{k}^{f}$ is the variance of it. Thus, the measurement $z_{k}$ can be expressed as

(20) $$z_{k}=H_{k}^{f}f+e_{k}^{f},\;e_{k}^{f}∼N\left(0,R_{k}^{f}\right).$$

In tracking application, the target extent $x_{k}^{f}$ is the instantiation of function value f, i.e., $f=x_{k}^{f}$. Similar to Equation (20), the radius value on the $\psi_{k,l}^{L}\left(x_{k}^{c},\zeta_{k}\right)$ direction can be expressed as

(21) $$f\left(\psi_{k,l}^{L}\left(x_{k}^{c},\zeta_{k}\right)\right)=H^{f}\left(\psi_{k,l}^{L}\left(x_{k}^{c},\zeta_{k}\right)\right)x_{k}^{f}+e_{k,l}^{f},$$

where $H^{f}\left(\cdot \right)$ denotes the observation model and $e_{k,l}^{f}$ denotes the noise of it. Substitute Equation (21) into Equation (19), the measurement equation can be rewritten as

(22) $$\begin{array}{cc} {\tilde{z}}_{k,l} & =x_{k}^{c}+p_{k,l}\left(x_{k}^{c}\right)\left[H^{f}\left(\psi_{k,l}^{L}\left(x_{k}^{c},\zeta_{k}\right)\right)x_{k}^{f}+e_{k,l}^{f}\right]+{\bar{e}}_{k,l}\\ & =\underset{\tilde{h}\left(x_{k}^{c},\zeta_{k},x_{k}^{f}\right)}{\underset{︸}{x_{k}^{c}+{\tilde{H}}_{l}\left(x_{k}^{c},\zeta_{k}\right)x_{k}^{f}}}+\underset{{\tilde{e}}_{k,l}}{\underset{︸}{p_{k,l}\left(x_{k}^{c}\right)e_{k,l}^{f}+{\bar{e}}_{k,l}}}\\ & ={\tilde{h}}_{k,l}\left(x_{k}\right)+{\tilde{e}}_{k,l}, \end{array}$$

where

(23) $${\tilde{H}}_{l}\left(x_{k}^{c},\psi_{k}\right)=p_{k,l}\left(x_{k}^{c}\right)H^{f}\left(\psi_{k,l}^{L}\left(x_{k}^{c},\zeta_{k}\right)\right),$$

(24) $$H^{f}\left(\psi_{k,l}^{L}\left(x_{k}^{c},\zeta_{k}\right)\right)=K\left(\psi_{k,l}^{L},\psi^{f}\right){\left[K\left(\psi^{f},\psi^{f}\right)\right]}^{-1},$$

(25) $$x_{k}\overset{\Delta }{\;=}\left(x_{k}^{c},\zeta_{k},x_{k}^{f}\right).$$

According to Equation (22), it is easy to obtain the new noise covariance as follows:

(26) $${\tilde{R}}_{k,l}=p_{k,l}R_{k,l}^{f}p_{k,l}^{T}+{\bar{R}}_{k,l},$$

(27) $$R_{k,l}^{f}=R^{f}\left(\psi_{k,l}^{L}\left(x_{k}^{c},\zeta_{k}\right)\right),$$

where $R_{k,l}^{f}$ is the covariance of noise $e_{k,l}^{f}$, and ${\bar{R}}_{k,l}$ is the covariance of noise ${\bar{e}}_{k,l}$.

### 3.3. Motion Model

We model the target state as $x_{k}={\left({\bar{x}}_{k},x_{k}^{f}\right)}^{T}$, where ${\bar{x}}_{k}=\left(x_{k}^{c},\zeta_{k}\right)$ is the centroid, $\zeta_{k}$ denotes the orientation of the extended target and $x_{k}^{f}$ is the extent. Because the target centroid is different from that of the extended, the centroid and the extent state are modeled separately. The dynamics of the target centroid ${\bar{x}}_{k}$ is consistent with the constant velocity model:

(28) $${\bar{x}}_{k+1}=\bar{F}{\bar{x}}_{k}+{\bar{\omega }}_{k}\;{\bar{\omega }}_{k}∼N\left(0,\bar{Q}\right),$$

where the transition matrix $\bar{F}$ and covariance matrix $\bar{Q}$ for target centroid ${\bar{x}}_{k}$ are given as

(29) F¯=1T01⊗I3,   Q¯=Ts3Ts333Ts2Ts222Ts2Ts222Ts⊗σq2000σq2000σqζ2,

where *T* denotes the sampling period, ⊗ denotes the Kronecker product, $I_{3}$ denotes the third order identity matrix, $\sigma_{q}^{2}$ and $\sigma_{q^{\zeta }}^{2}$ represents the process variance of the centroid and orientation. The dynamics of target extent $x_{k}^{f}$ is shown as follows:

(30) $$x_{k+1}^{f}=F^{f}x_{k}^{f}+{\omega^{f}}_{k}\;{\omega^{f}}_{k}∼N\left(0,Q^{f}\right),$$

where the extent transition matrix and covariance matrix are presented as

(31) $$F^{f}=\exp\left(-\alpha T\right),$$

(32) $$Q^{f}=\left(1-\exp\left(-2\alpha T\right)\right)\cdot K\left(\psi^{f},\psi^{f}\right),$$

where α is the forgetting factor.

Finally, transition model of the augmented state, i.e., the combination of dynamics of target state ${\bar{x}}_{k}$ and extent $x_{k}^{f}$, is given as

(33) $$x_{k+1}=Fx_{k}+\omega_{k}\;\omega_{k}∼N\left(0,Q\right),$$

where

(34) $$\begin{array}{ccc} x_{k}={\left[{\bar{x}}_{k},x_{k}^{f}\right]}^{T} & F=\left[\begin{array}{cc} \bar{F} & 0\\ 0 & F^{f} \end{array}\right] & Q=\left[\begin{array}{cc} \bar{Q} & 0\\ 0 & Q^{f} \end{array}\right]. \end{array}$$

The initial state $x_{0}$ is given as

(35) $$\begin{array}{cc} x_{0}\left(\mu_{0},P_{0}\right),\;\mu_{0}={\left[{\bar{\mu }}_{0},0\right]}^{T} & P_{0}=\left[\begin{array}{cc} {\bar{P}}_{0} & 0\\ 0 & P_{0}^{f} \end{array}\right], \end{array}$$

where ${\bar{\mu }}_{0}$ and ${\bar{P}}_{0}$ denote the mean and covariance of ${\bar{x}}_{k}$, $P_{0}^{f}$ denotes the covariance of $x_{k}^{f}$, which can be calculated by Equation (22).

So far, the state space description is provided in Equations (22) and (33). Now, the estimation can be performed using a nonlinear filter and EKF is employed in this letter. The calculation of Jacobian matrix is deeply discussed in the appendix of Ref [18].

## 4. Simulation and Analysis

### 4.1. Simulation Setting

**Figure 2:**
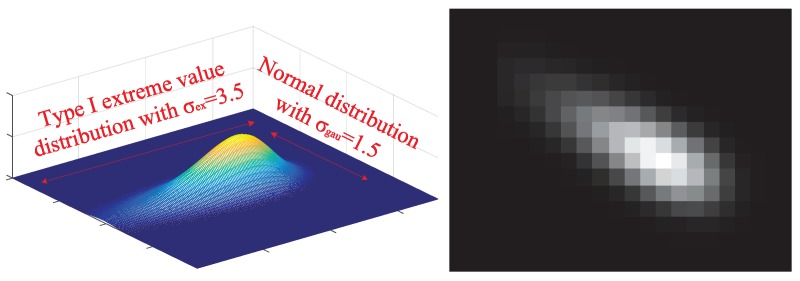
A view of the target model and its projection on the image plane.

An extended target with grayscale information moves in the [300×300] surveillance area. The total number of scans is set to 100 s and the scan cycle is set to $T_{s}=1$ s. The grayscale amplitude along the velocity direction of the target follows the type I extreme value distribution with the scale parameter $\sigma_{ex}$. The grayscale amplitude on a direction perpendicular to velocity obeys the Gaussian distribution with the standard deviation $\sigma_{gau}$. A view of the amplitude distribution model of the target is shown in Figure 2.

The standard deviations of the process noise on position and angle are set to be $\sigma_{q}\;=1$ and $\sigma_{q^{\zeta }}\;=\;0.1$, respectively, forgetting factor α=0.0001. The standard deviations of the measurement noise on position and amplitude are set as $\sigma_{r}\;=\;1$ and $\sigma_{I}\;=\;0.1$. The hyper-parameter of the GP are set to $\sigma_{f}=2$, $\sigma_{r}=2$ and l=ππ44. Those pixels with amplitude value greater than $Th_{gray}=0.01$ are retained as target measurements *z* and the others are removed. Each simulation are tested within 50 Monte Carlos.

### 4.2. Feature Estimation Steps

**Figure 3:**
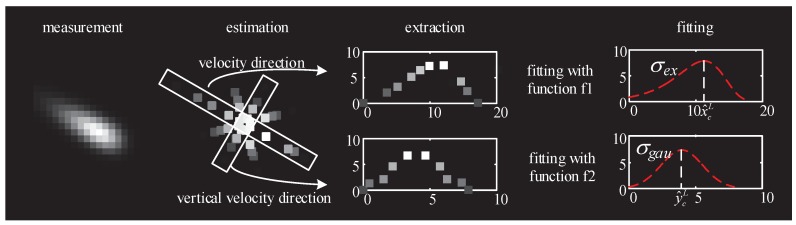
Flow chart of feature extraction.

In addition to the estimation of the target centroid $x_{k}^{c}$, another important goal is to extract the target feature parameters based on the estimated values $x_{k}^{f}$ at all directions. In this study, the steps shown in Figure 3 are designed to extract the target distribution feature parameters $\sigma_{ex}$ and $\sigma_{gau}$. In this feature estimation method, the estimated points along velocity direction and vertical velocity direction are extracted. These points are fitted with function $f_{1}$ and $f_{2}$, which is given below. After the fitting step, the center estimation $\left({\hat{x}}_{c}^{L},{\hat{y}}_{c}^{L}\right)$ and feature parameters $\sigma_{ex}$ and $\sigma_{gau}$ are easily attained.

For the estimation step, too many $\psi^{f}$ will increase the calculation cost and cause the irreversibility of the covariance function $K(\psi^{f},\psi^{f})$. However, if the number of input is small, the estimation accuracy of target extent cannot be guaranteed. As a compromise, we set the input number as follows. For the proposed GP with AI, eight points are uniformly sampled within the interval of $\left[0∼2\pi \right]$ as azimuth $\theta^{f}$ and five points are uniformly sampled within the interval of $\left[0∼\pi /2\right]$ as $\varphi^{f}$. For the traditional GP, eight points are uniformly sampled within the interval of $\left[0∼2\pi \right]$. Since the number of Fourier coefficients in RHMs has to be odd, and to ensure that the number of parameters to be evaluated is consistent with that of GP as much as possible, we set the coefficients number to 9.

As for the extraction and fitting step, the feature parameters $\sigma_{ex}$ and $\sigma_{gau}$ can be subsequently obtained by fitting the estimated points on velocity direction and vertical velocity direction. Since the distribution of the trailing target on velocity and vertical velocity direction follows the type I extreme value distribution and Gaussian distribution, the functions $f_{1}$ and $f_{2}$ are given as

(36) $$f_{1}=A_{1}\cdot \underset{type\;I\;extreme\;value\;distribution}{\underset{︸}{\frac{1}{\sigma_{ex}}\cdot \exp\left(\frac{x-\mu_{ex}}{\sigma_{ex}}\right)\cdot \exp\left(-\exp\left(\frac{x-\mu_{ex}}{\sigma_{ex}}\right)\right)}},$$

(37) $$f_{2}=A_{2}\cdot \underset{Gaussian\;distribution}{\underset{︸}{\frac{1}{\sqrt{2\pi }\sigma_{gau}}\cdot \exp\left(\frac{{\left(x-\mu_{gau}\right)}^{2}}{\sigma_{gau}^{2}}\right)}}.$$

Subsequently, ${\hat{x}}_{c}^{L}$ and ${\hat{y}}_{c}^{L}$, corresponding to the maximum value on each fitting curve in Figure 1, denotes the target local coordinate position, respectively.

### 4.3. Shape Deformation

**Figure 4:**
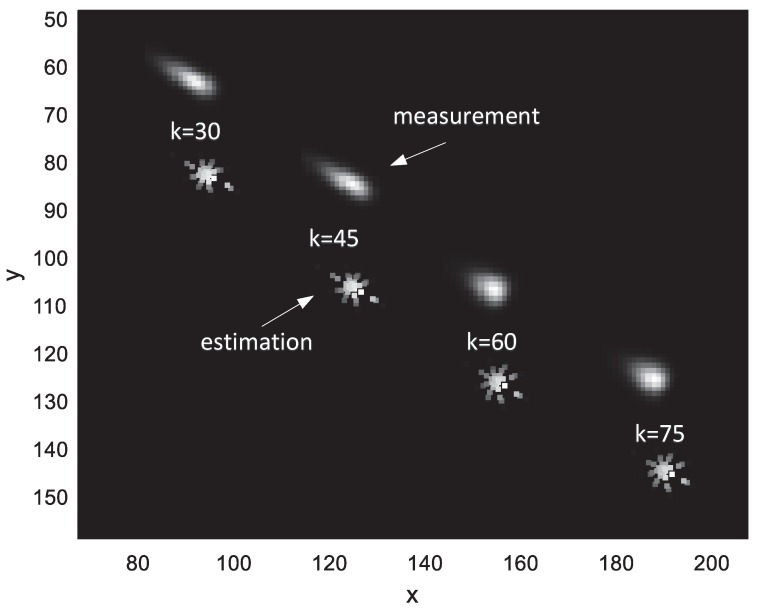
Snapshots of tracking at time step k = 30, 45, 60 and 75.

The target moves at a constant speed and the initial state of the target centroid is ${\left[31,23,1.2,1.1\right]}^{T}$. The scale parameter $\sigma_{ex}=3.5$ and becomes 2.5 at the 50th second. The standard deviation $\sigma_{gau}=1.5$ and becomes 2 at the 50th second. The sum of the target grayscale is 200. The performance is measured with the tracking precision and target feature parameter estimation accuracy. Figure 4 shows a snapshot of the tracking process. For display convenience, we added a bias of 20 pixels on the *y*-axis of the estimation. It can be observed that the shape estimate changes with the measurement progressively.

**Figure 5:**
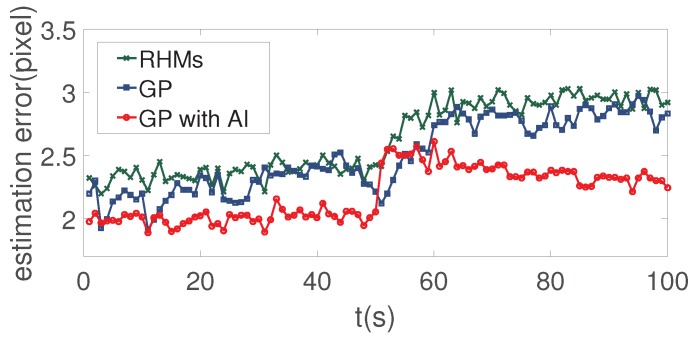
Comparison of tracking precision between RHMs, GP and the proposed GP with AI.

**Figure 6:**
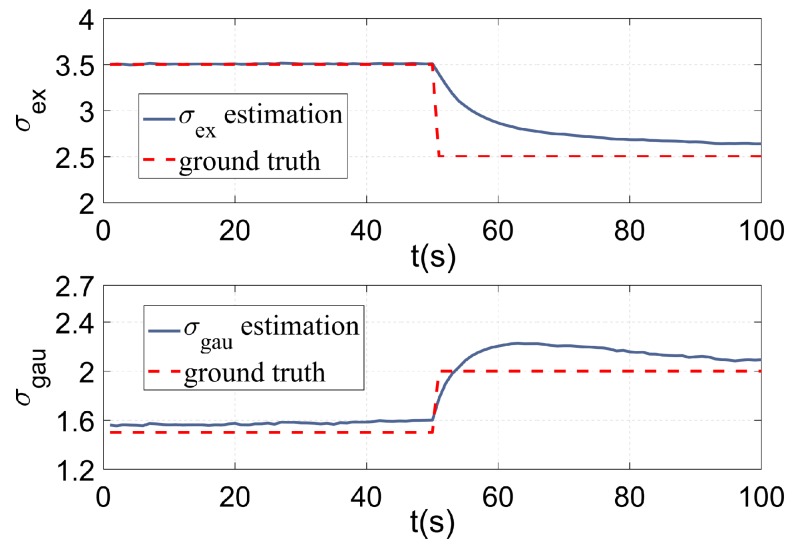
Estimation of $\sigma_{ex}$ and $\sigma_{gau}$ vs. ground truth for a shape deformation scenario.

Figure 5 compares the tracking precision of the RHMs, GP, and the proposed GP with AI. It can be seen from Figure 5 that the proposed method outperforms the RHMs and GP method. Because the RHMs and the traditional GP method can only use the position information of the measurements, the contribution of each pixel to the target centroid is the same, so traditional methods can only estimate the center of form. On the other hand, the proposed GP with AI can estimate the center of mass with the amplitude information introduced. The estimation of target feature $\sigma_{ex}$ and $\sigma_{gau}$ are given in Figure 6, the red dotted lines denote the ground truth of target features and the blue lines denote the estimation of target features. It can be seen that the proposed method estimates the ground truth quite well during the first 50 s. Even if the target extent mutates in the 50th second, the proposed method is able to approximate the ground truth gradually with the new measurements’ accumulation. The result demonstrates that the proposed method has the ability to adapt to the shape-shifting extended target. There exists deviation between the estimation and the ground truth value since the systematic error is brought in by the truncation of measurement amplitude by $Th_{gray}$ and the fitting step.

### 4.4. Motion Change

**Figure 7:**
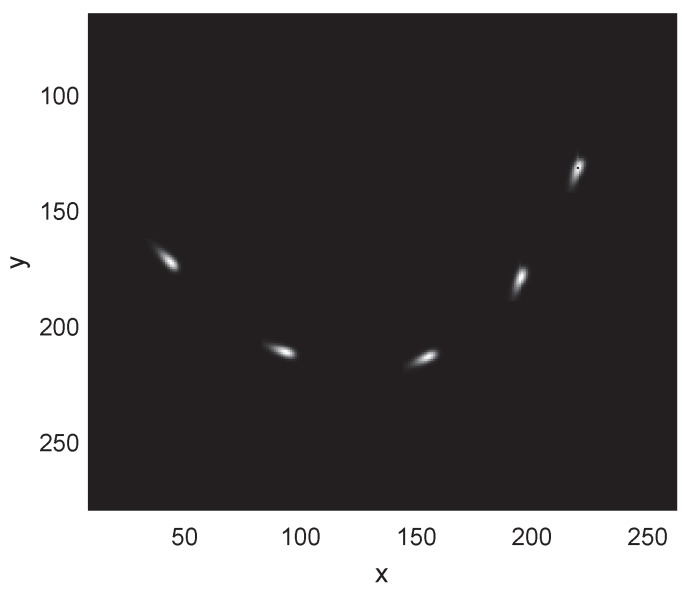
Snapshots of target motion with turn rate ω=π/80.

**Figure 8:**
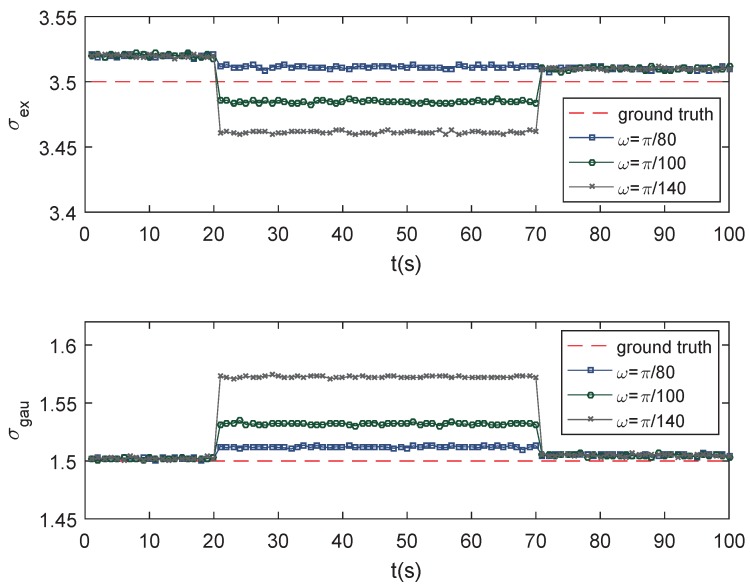
Estimation of $\sigma_{ex}$ and $\sigma_{gau}$ with a different turn rate.

The scale parameter $\sigma_{ex}=3.5$ and standard deviation $\sigma_{gau}=1.5$. The sum of target grayscale is 200. The target motion model follows Equation (38) during the 20th to 70th second and constant speed model during the rest of the simulation time

(38) $$F=\left[\begin{array}{cccc} 1 & 0 & \sin(\omega T_{s})/\omega & -(1-\cos(\omega T_{s}))/\omega \\ 0 & 1 & (1-\cos(\omega T_{s}))/\omega & \sin(\omega T_{s})/\omega \\ 0 & 0 & \cos(\omega T_{s}) & -\sin(\omega T_{s})\\ 0 & 0 & \sin(\omega T_{s}) & \cos(\omega T_{s}) \end{array}\right],$$

where ω is the turn rate. Figure 7 shows a snapshot of a target with turn rate ω=π/80. Figure 8 shows the feature parameter estimation results of the targets moving with ω=π/80, π/100 and π/140, respectively.

It can be seen that the feature parameter estimation performance goes worse with the increase of the target maneuverability.

### 4.5. Intensity Change

**Figure 9:**
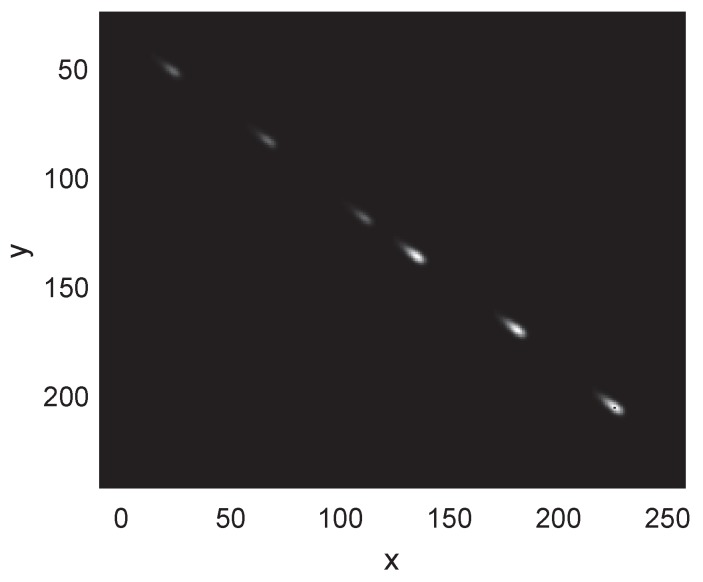
Snapshots of a tracking scene of target intensity change.

**Figure 10:**
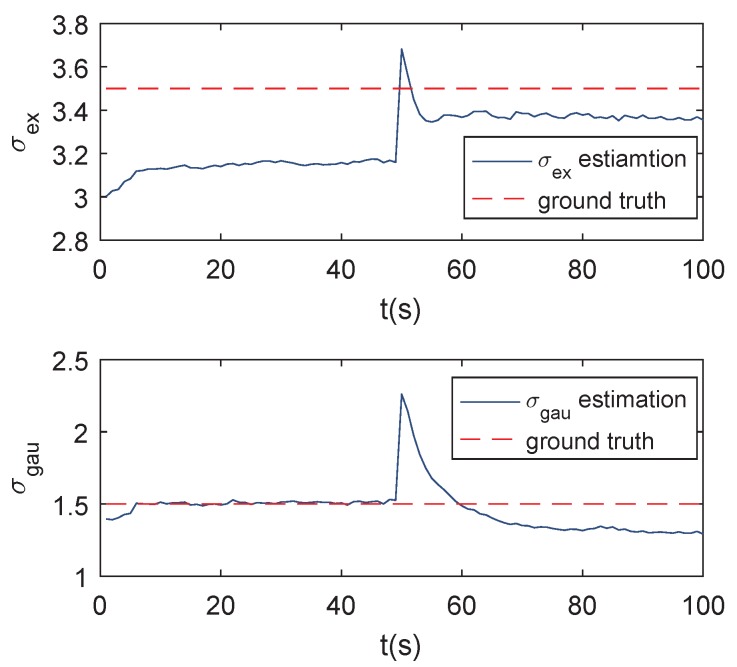
Estimation of $\sigma_{ex}$ and $\sigma_{gau}$ for an intensity change scenario.

The scale parameter $\sigma_{ex}=3.5$ and standard deviation $\sigma_{gau}=1.5$. The sum of target grayscale changes from 200 to 500 at the 50th second. Figure 9 shows a snapshot of intensity change and Figure 10 shows the feature estimation results.

It can be seen that, even if the target parameters’ estimation has a large deviation as the intensity abrupt changes at the 50th second, it tends to converge to the ground truth as long as the intensity of the target holds steady.

## 5. Conclusions

In this letter, a GP based method for the tracking of an extended target by using amplitude information is proposed. Surface shape and kinematic state are simultaneously estimated. The superiority of utilizing the amplitude information is demonstrated compared with traditional RHMs and GP methods. As a result, the tracking error implemented by the proposed method reduces about 0.5 pixels compared with the RHMs and GP. Furthermore, by using the estimated points on the target surface, we also extract the target feature parameters, which can be used for target classification in further study. The estimation error of feature parameters $\sigma_{ex}$ and $\sigma_{gau}$ are able to converge to 0.17 and 0.1, respectively. Through challenging scenes in Section 4.4 and Section 4.5, the estimation performance can be limited, influenced by the mutations on target maneuvering and intensity changing, which proves the robustness of our proposed method. In fact, the tracking performance improvement is mainly due to the introduction of amplitude information. If the amplitude information is ignored, this method degrades to the traditional GP method, and its performance will be no different from that of the GP and RHM methods. Since the proposed method does not require any parametric model, it is flexible enough to estimate any convex hemisphere-shaped target.
